# Notes on Visual Cortical Feedback and Feedforward Connections

**DOI:** 10.3389/fnsys.2022.784310

**Published:** 2022-01-28

**Authors:** Kathleen S. Rockland

**Affiliations:** Department of Anatomy and Neurobiology, School of Medicine, Boston University, Boston, MA, United States

**Keywords:** apical dendrites, hierarchy, layer 1, looped neurons, reciprocity, pyramidal subtypes

## Introduction

Feedforward (FFD) – feedback (FBK) cortical processing ultimately needs to be considered in the context of whole-brain activation, including interactions with cortico-thalamo-cortical, callosal, and the excitatory and inhibitory intrinsic cortical circuits. For the non-human primate (NHP) brain, however, identifying cell types and deciphering the patterns and metrics of axon convergence and divergence is challenging (cf. Rockland, 2019, 2020) and, at the level of detail approachable in the mouse brain, may still be years away. Many of the comments put forth here are not novel and echo previous reports (including my own, Rockland, 1997). My goal has been to briefly re-consider what have become key features of FFD-FFK connections in the early visual cortical pathway, with emphasis on the cellular and dendritic circuitry components. Owing to sparsity of data in NHP concerning the role of interneuron subpopulations in microcircuitry, these are not discussed. For detailed reports on visual cortical connectivity and physiological response properties (see Bullier, 2004; Douglas and Martin, 2007; Shipp, 2007, 2016; Markov et al., 2014a,b; Angelucci et al., 2017; Vanni et al., 2020; Vezoli et al., 2021, among others).

Although area V1 is a canonical “start point” for discussing FFD-FBK cortical processes, it is actually something of an outlier; that is, there are cortico-thalamic, but not cortico-cortical FBK projecting neurons in V1; and FFD terminations are of thalamic, but not cortical origin. There are few or no callosal connections. Thus, a strict comparison of *cortical* FFB and FBK connections is better addressed in extrastriate areas V2, V3, V4, MT, or TEO. Much of the following discussion is written as applying to V2.

### Neurons of Origin

**FIGURE 1:**
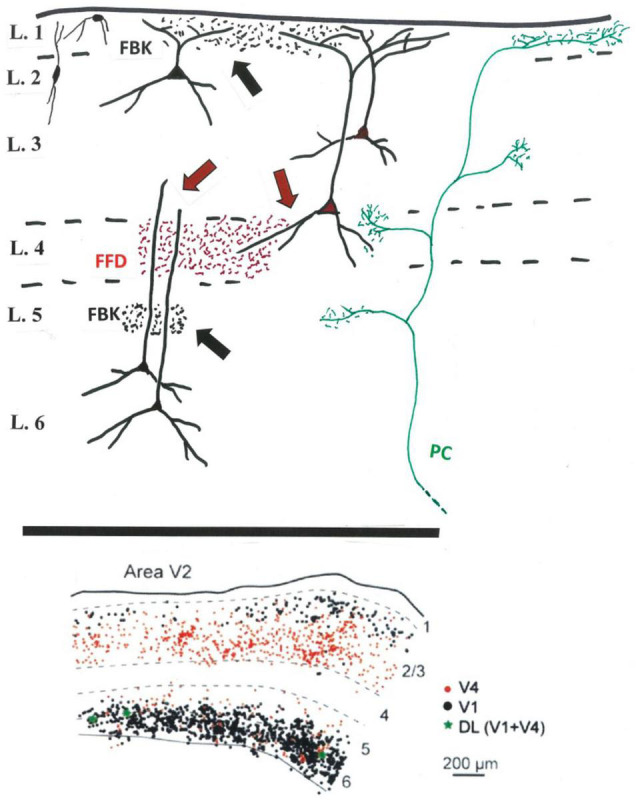
Above: Schematic sketch (V2) of FFD (in red) and FBK terminations (in black) in relation to potential postsynaptic dendrites (black) of FFD (red soma) and FBK (black soma) projecting neurons. FFD teminations in layer 4 (red arrows) potentially contact small pyramidal cells or interneurons in layer 4, basal dendrites of FFD neurons, and distal dendrites of FBK neurons. FBK or other terminations in the infragranular layers (black arrow) might access more proximal apical dendrites of infragranular neurons. Included for reference, a typical, multilaminar pulvino-cortical (PC) axon (in green) (And see Federer et al., 2021: terminations in V1 after viral infection of V2). Not shown: layer 5 neurons, inhibitory neurons (except for the representative neurons at upper left), intra- and interlaminar intrinsic connections, and the additional afferent inputs to layer 1 or other layers. Below: Predominant segregation of FFD (in red) and FBK (in black) projecting neurons in V2, as demonstrated by double retrograde injections in V1 and V4, with a small number (in green) of double labeled (DL) neurons (modified from Figure 10 in Markov et al., 2014b).

As repeatedly summarized in the literature (e.g., Kennedy and Bullier, 1985: Rockland, 1997, 2019; Douglas and Martin, 2007; Markov et al., 2014a,b; Anderson and Martin, 2016; Angelucci et al., 2017, among others), FFD projecting neurons from V2 (to V4 and MT) and FBK projecting neurons (to V1) are differentially located in deeper layer 3 (FFD) or layers 2, 3A, 5, and 6 (FBK). The FFD-FBK laminar dissociation, despite a minor degree of laminar intermingling, has been largely confirmed by injections of two distinguishable retrograde tracers in V1 and V4 (Markov et al., 2014b; and see Figure 1), where less than 1% of cortically projecting cells in V2 (and 2.2% in V3) were double labeled (i.e., had branching collaterals to both V1 and V4). The further characteristics of these bifurcating, link neurons, and their postsynaptic targets, are unknown. Are they more frequent in the less investigated peripheral visual representation of V2, or for other combinations (e.g., injections in V1 and MT)?

The most numerous FBK population is in layer 6 (see estimates in Table 3 in Rockland, 1997; Markov et al., 2014a,b). Along with the smaller number of layer 5 FBK neurons, this infragranular distribution overlaps with that of several cortico-subcortical projecting populations (cortico-collicular, cortico-striatal, or cortico-thalamic projecting neurons in layer 5, and cortico-thalamic or cortico-claustral neurons in layer 6; summarized in Shipp, 2007). Appropriate double retrograde tracer experiments have not been done to probe for collateralization of cortico-cortical and cortico-subcortical axons. Whether these neuronal subpopulations are spatially clustered or distributed in a salt-and-pepper pattern has not been established (but see Hawken et al., 2020 for “functional clusters” in V1).

### Neuronal Subtypes

Feedback and feedforward neurons are excitatory pyramidal neurons, although a small number of GABAergic FBK neurons, probably positive for nitric oxide or somatostatin, are found in the supragranular layers of V2 after viral infection in V1 (Tomioka and Rockland, 2007). Pyramidal subtypes can be more finely distinguished, in part by dendritic morphology. Supragranular neurons extend their apical dendrite into layer 1. For layer 6 neurons and many layer 5 FBK neurons, the apical dendrite extends only into layer 3. A subset of layer 5 neurons send apical dendrites to layer 1 (Golgi stains: Lund et al., 1981); and intracellular fills of tracer identified FFD projection neurons demonstrated about half (4 of 9 neurons) having apical dendrites that extend to layer 1 (Markov et al., 2014b). Soma depth is significant, in that shorter apical dendrites, even of neurons in the same layer, are reported to be less excitable (in mice: Galloni et al., 2020).

By comparison, five subtypes of morphologically distinct cortico-geniculate (CG) neurons in V1 and at least three subtypes in V2 have been identified (Briggs et al., 2016). Heterogeneity of CG neurons is supportive of some degree of parallel processing (“…not one circuit, but rather a collection of distinct circuits conveying unique [visual] feature information and operating on a corresponding variety of timescales”; Briggs, 2020); and this may apply as well to FFD and FBK processes (“…a multiplicity of feedback pathways involved in a wide range of cognitive functions”; Vezoli et al., 2021).

Other anatomical evidence of neuron diversity includes input diversity (for V1: Sawatari and Callaway, 2000); soma size [FBK neurons in layer 6, but not necessarily layer 5, are smaller (Rockland, 2004; Berezovskii et al., 2012)], and the observation that some but only some layer 6 FBK neurons use synaptic zinc (Ichinohe et al., 2010), an activity related neural modulator (McAllister and Dyck, 2017). Some but only some neurons in V4, TEO, or MT branch to both V2 and V1, as demonstrated by single axon reconstructions (Rockland et al., 1994; Rockland and Knutson, 2000), and by double retrograde tracer injections in V1 and V2 (Kennedy and Bullier, 1985). Transcriptomic investigations are likely to reveal further criteria of diversity, as already reported for neurons in layer 6 of V1 (Hawken et al., 2020; and proposed as a general rule: Cembrowski and Spruston, 2019).

Axon data are more sparse than data for dendrites, but differences in myelination, axon caliber, and the topology of the distal arbors are consistent with there being multiple neuronal subpopulations (see comments in Rockland, 2020). Area V2 axons (laminar source not known) terminating in V1 are (1) slender (0.3 μm in diameter), unbranched, unmyelinated, and uniformly covered with boutons terminaux, or (2) thick (>1.0 μm), branched, heavily myelinated, and forming separate small clusters of large, multisynaptic boutons (Anderson and Martin, 2009). Divergent and/or clustered terminal arborizations are similarly reported by reconstructions of single axons projecting to V1 from V2 (Rockland and Virga, 1989) or from MT (Rockland and Knutson, 2000). Some V4 axons are reported to terminate in V2 with clustered boutons between myelinated lengths, while others are unbranched and have a continuous distribution of boutons with no intercalated myelin (Anderson and Martin, 2006).

### Axon Interactions

Feedback and feedforward connections are part of a rich nexus that includes thalamo-cortical, callosal, amygdalo-cortical (Freese and Amaral, 2005), claustro-cortical, excitatory intrinsic collaterals, local inhibitory terminations, and neuromodulatory projections (Kravitz et al., 2013; Rockland, 2019, 2020; Vanni et al., 2020, and further references therein). Inactivation experiments provide evidence that FBK connections have specific functional influence, but not how this comes about. Physiological perturbations have been demonstrated in V2 following separate inactivation of areas V4, MT, or pulvinar (Correia et al., 2021; and for V2 to V1: Nassi et al., 2013; Nurminen et al., 2018). Putative interaction with excitatory intrinsic connections, to give another example of FBK influences, has been documented by simultaneous recordings in V1 and V4. Contour-related neuronal responses are found to emerge initially in V4, following ∼40 ms later in V1, and then continuing to develop in parallel in both areas (Chen et al., 2014). This was proposed as an incremental process, where visual contour information accumulates in parallel over multiple areas, presumably both cortical and subcortical. This process could be carried out by direct FBK from V4 to V1, a polysynaptic V4-V2-V1 routing, and/or interactions of FBK signals with horizontal intrinsic connections in V1 (Liang et al., 2017).

What are the cellular and microcircuitry substrates of FFD-FBK processes? Relevant anatomical data are woefully lacking, as surveyed above, and answers remain on the order of what we’d like to know. This includes:

(1) Specific data on intrinsic inter- and intralaminar pyramidal cell collaterals, for identified FBK and FFD projecting neurons. From intracellular fills (in cat: Gilbert and Wiesel, 1983; Martin and Whitteridge, 1984), these are known to be spatially extensive, with hundreds to thousands of terminations, and can be inferred to converge, within and across layers, with multiple extrinsic connections and other collaterals. The range of collaterals (2–12 per neuron?) and degree of neuron-to-neuron variability is unknown, although excitatory intrinsic terminations are recognized to be the numerically major synaptic subpopulation (80% of the total), as opposed to any of the extrinsic cortical or subcortical connections (e.g., Anderson and Martin, 2016).
(2) What are the postsynaptic targets: do FFD and FBK projecting neurons preferentially target other FFD and FBK neurons? Always or in what proportion? Does this differ topographically within areas or across different areas? Electron microscopic (EM) investigations in NHP establish that FFD terminations from V1 to V2, FBK terminations from V2 to V1, and FBK from V4 to V2 target both GABergic profiles (∼14%) and dendritic spines (Anderson and Martin, 2006, 2009). FFD axons from V2 to MT terminate on dendritic spines in layer 4 and layers 1, 2 (respectively, 67 and 82% of the postsynaptic pool; Anderson and Martin, 2002). The dendritic spines presumably belong to pyramidal neurons, but which neurons, and is there functionally significant synaptic clustering (for ferret: Scholl et al., 2021)?
(3) Dendritic location and pattern of identified synapses. Calcium imaging allows visualization of individual synapses on identified dendrites and is beginning to provide spatiotemporal synaptic maps. Orientation- and chromatic-selective inputs have been mapped for superficial pyramidal neurons in V1, with evidence of a wide scattering of functional properties, perhaps reflecting dendritic integration within and across visual feature domains (Ju et al., 2020). Further results are needed at this level of resolution.

### Feedforward-Feedback Reciprocity

Area-to-area reciprocity has been a hallmark feature of FFD-FBK processes (e.g., predictive coding Shipp, 2016; Pennartz et al., 2019), and reciprocity has recently been extended to investigations at the level of neuron-to-neuron. Viral mediated monosynaptic circuit tracing demonstrates FBK inputs from V2 to some V1 neurons that send FFD projections to V2 (i.e., “looped neurons,” Siu et al., 2021). The frequency of such neuron-to-neuron loops is not yet known, nor the specific details of synaptic number and location. FBK axons have hundreds of terminations, of which only a small, and presumably variable number (1–10?) contact any single neuron. Thus, an important aspect of neuron-to-neuron reciprocity is how this is elaborated in relation to a putative assembly of multiple locally adjacent neurons, many of which are likely to be themselves directly and indirectly interlinked by the network of intrinsic collaterals.

Feedback axons have repeatedly been reported as spatially divergent (Rockland, 1997; Anderson and Martin, 2016; Vezoli et al., 2021) with a spatially asymmetric axonal distribution in relation to the territory occupied by retrogradely labeled FFD cells (“reciprocal asymmetry,” Shipp and Zeki, 1989). Divergent FBK axons often carry small clusters of terminations, and this “hybrid” spatial distribution might indicate a combination of topographically reciprocal and asymmetrical connections (Rockland and Virga, 1989; Angelucci et al., 2017).

Area-to-area reciprocity, despite the attractiveness of the idea, is evidently not an obligatory feature of FFD-FBK connections. There are cortical connections which would be considered as FBK (i.e., not projecting to layer 4), but which are not reciprocated; namely, unidirectional projections to V1 from TEO, TE, TF, and TH (Kennedy and Bullier, 1985; Rockland and Van Hoesen, 1994; Suzuki et al., 2000; Kravitz et al., 2013; Markov et al., 2014a), and there are unidirectional projections to the peripheral field representations of V1 and V2 from auditory (Falchier et al., 2002; Rockland and Ojima, 2003) or parietal areas (Borra and Rockland, 2011). “Leapfrog” connections have been identified in the FFD visual pathway (V2 to TEO; summarized in Kravitz et al., 2013).

### Laminar Signatures

From the perspective of presynaptic and postsynaptic neuropil, the distinction between FBK-dominated layer 1 and FFD-dominated layer 4 is not clearcut (Figure 1). The soma location is only a provisional predictor of segregated dendritic inputs distant from the soma. That is, apical dendrites of both supragranular FFD and supragranular FBK neurons access potentially common inputs in layer 1. Input to layer 4 is accessible to both FFD and FBK neurons, but at different dendritic locations; namely, basal dendrites of FFD projecting neurons in lower layer 3, or distal apical dendrites for infragranular FBK neurons, in addition to indirect interlaminar relays from layer 4 neurons potentially to both populations.

There is also common within-area axon collateralization across multiple layers. In V2, pulvinocortical axons (“FFD” by analogy with geniculocortical axons in V1) are typically multilaminar (Rockland et al., 1999). FFD axons from V2 to MT terminate in layers 1 and 4 (Anderson and Martin, 2002). FBK axons from both V2 and MT to V1 frequently have collaterals, usually in layer 5 (Rockland, 1997; Anderson and Martin, 2016). The relative frequency of multilaminar collateralization of a single axon and the postsynaptic targets are not known.

### Cortical Networks and Hierarchy

In this Opinion, I have shared my view of “what we’d like to know” or, more precisely, what we need to know for better understanding of functional organization (also, Rockland, 2019, 2020). This is in part (1) more detail (better definition of cell types, more data on microcircuitry) but also (2) a broader context, of how FFD and FBK processes interact with multiple extrinsic and intrinsic connections under different conditions.

The FFD and FBK architecture has been closely associated with cortical hierarchy, serving to some extent as a proxy of rank-ordering. The nature of “hierarchy” itself, however, continues to generate discussion (e.g., Pessoa, 2018; Hilgetag and Goulas, 2020; and for recent review of rodent and NHP: Gamanut and Shimaoka, 2021). Other ideas have been raised: parallel streams of hierarchical processing that overlap in space and time (Lamme and Roelfsema, 2000); multiregional coordination (“coordination dynamics,” Tognoli and Kelso, 2014); hierarchical heterogeneity of cross-area intrinsic local properties (Demirtas et al., 2019); multiple, parallel and asynchronously operating task- and stimulus dependent hierarchies (Zeki, 2016, 2020), among others. As suggested almost twenty years ago, “different cues are processed with different priorities and interact in a complex fashion [such that] processing involves many areas of the hierarchy at the same time, with information flowing in the feedforward as well as the feedback direction” (Bullier, 2004).

## Author Contributions

The author confirms being the sole contributor of this work and has approved it for publication.

## Conflict of Interest

The author declares that the research was conducted in the absence of any commercial or financial relationships that could be construed as a potential conflict of interest.

## Publisher’s Note

All claims expressed in this article are solely those of the authors and do not necessarily represent those of their affiliated organizations, or those of the publisher, the editors and the reviewers. Any product that may be evaluated in this article, or claim that may be made by its manufacturer, is not guaranteed or endorsed by the publisher.
